# Decoding brain structure to stage Alzheimer's disease pathology in Down syndrome

**DOI:** 10.1002/alz.14519

**Published:** 2025-01-14

**Authors:** James T. Kennedy, Julie K. Wisch, Aylin Dincer, June Roman, Brian A. Gordon, Benjamin Handen, Tammie L. S. Benzinger, Elizabeth Head, Mark Mapstone, Bradley T. Christian, Dana L. Tudorascu, Charles L. Laymon, Sigan L. Hartley, Patrick Lao, Adam M. Brickman, Shahid H. Zaman, Beau M. Ances, the ABC‐DS and DIAN Consortia

**Affiliations:** ^1^ Department of Neurology Washington University School of Medicine St. Louis Missouri USA; ^2^ Department of Radiology Washington University School of Medicine St. Louis Missouri USA; ^3^ Department of Psychological & Brain Sciences Washington University St. Louis Missouri USA; ^4^ Department of Psychiatry University of Pittsburgh Pittsburgh Pennsylvania USA; ^5^ Department of Pathology and Laboratory Medicine University of California Medical Sciences D Irvine California USA; ^6^ Department of Neurobiology and Behavior University of California Irvine California USA; ^7^ Department of Neurology University of California Irvine California USA; ^8^ Waisman Laboratory for Brain Imaging and Behavior University of Wisconsin‐Madison Madison Wisconsin USA; ^9^ Department of Medical Physics University of Wisconsin‐Madison Madison Wisconsin USA; ^10^ Department of Radiology University of Pittsburgh Pittsburgh Pennsylvania USA; ^11^ Department of Human Development & Family Studies University of Wisconsin‐Madison Madison Wisconsin USA; ^12^ Department of Neurology Columbia University New York New York USA; ^13^ Gertrude H. Sergievsky Center and Taub Institute for Research on Alzheimer's Disease and the Aging Brain Columbia University New York New York USA; ^14^ Cambridge Intellectual and Developmental Disabilities Research Group University of Cambridge Cambridge UK

**Keywords:** Alzheimer's, amyloid, autosomal dominant Alzheimer's disease, cognitive impairment, cortical thickness, dementia, Down syndrome, MRI, subcortical volume

## Abstract

**INTRODUCTION:**

Alzheimer's disease (AD) in Down syndrome (DS) is associated with changes in brain structure. It is unknown if thickness and volumetric changes can identify AD stages and if they are similar to other genetic forms of AD.

**METHODS:**

Magnetic resonance imaging scans were collected for 178 DS adults (106 nonclinical, 45 preclinical, and 27 symptomatic). Cortical thickness and subcortical volumes were compared between DS groups and evaluated as a staging metric using receiver operating characteristic analyses. Thickness patterns were compared to those previously reported in autosomal‐dominant AD (ADAD).

**RESULTS:**

Decreased parietal and temporal lobe thickness differentiated amyloid positivity (area under the curve [AUC] = 0.83) and impairment (AUC = 0.81), and slightly outperformed subcortical volumes (AUC = 0.8/0.74). Thickness differences in DS were more widespread, severe, and had better discriminative ability than ADAD.

**DISCUSSION:**

Cortical thickness can stage AD pathology in DS. Identification of brain regions affected by AD may aid in tracking disease course and evaluating treatment effects.

**Highlights:**

- DSAD is associated with reduced temporal and parietal cortical thickness.
- DSAD is associated with smaller hippocampal and striatal volumes.
- Thickness differences can stage DSAD better than other forms of AD.
- DSAD thickness differences are more extensive and severe than ADAD.

## 1 BACKGROUND

Alzheimer's disease (AD) is a debilitating neurological condition characterized by accumulation of amyloid‐beta (Aβ) plaques, tau tangles, neurodegeneration, and cognitive decline.^1^ Rare genetic forms of early‐onset AD have been identified, with Down syndrome (DS) AD (DSAD) and autosomal‐dominant AD (ADAD) being the most common genetically determined variants.^2^, ^3^ DSAD results from the triplication of the amyloid precursor protein (APP) and other genes on chromosome 21, and leads to AD neuropathology in the 40s and dementia in the 50s.^3^ ADAD arises from mutations in the APP, PSEN1, or PSEN2 genes, resulting in dementia onset around the age of the affected parent, typically in the 40s‐50s.^4^ Both DSAD and ADAD involve early accumulation of amyloid, with AD pathology evident ∼10‐20 years prior to clinical symptoms.^5^ These genetic forms of AD provide valuable insights into AD progression that may have broader implications. Examining magnetic resonance imaging (MRI) biomarkers may assist with early diagnosis and aid in identifying the regions affected along the AD continuum. These biomarkers could guide future AD clinical trials in DS.^6^

MRI allows for non‐invasive evaluation of pathological changes in cortical thickness and brain volumes. Cortical signatures, indicating group differences in thickness in specific brain regions, have been identified for late‐onset Alzheimer's disease (LOAD)^7^, ^8^, ^9^ and ADAD.^9^ LOAD is associated with a reduction in temporal and parietal lobes while ADAD is associated with thinning of the parietal lobe.^9^ Prior DS research identified decreases in posterior parietal, temporal, and occipital cortical thickness with AD pathology, but have not been evaluated along the AD continuum.^10^, ^11^, ^12^, ^13^, ^14^ Subcortical volume loss has been observed in ADAD, DSAD, and LOAD.^10^, ^12^, ^15^, ^16^ ADAD is associated with striatal and hippocampal volume loss.^15^ Previous studies in LOAD primarily focused on reductions in hippocampal volume with advancing AD.^16^, ^17^, ^18^ In DSAD, volume reductions are observed in the striatum, thalamus, and hippocampus.^10^, ^12^ Thresholds used to evaluate AD in ADAD and LOAD, especially for the hippocampus, may misclassify DSAD due to neurodevelopmental differences found in DS.^19^ Therefore, it remains uncertain what subcortical regions and thresholds are effective for identifying AD pathology in DS.

This study investigated neurodegenerative differences due to different stages of DSAD. Our analysis focused on identifying thickness and volume differences in both preclinical (cognitively stable, Aβ positive) and symptomatic (cognitively impaired, Aβ positive) AD. We hypothesized that a thinner parietal cortex^10^, ^11^, ^12^, ^13^ and smaller hippocampal volume would distinguish AD stages, with hippocampal atrophy outperforming cortical thinning^17^ based on the historically poor performance of cortical thickness measures in (LOAD) and ADAD. ^8^ We used the same preprocessing and analytical approach in developing our cortical signatures as previous ADAD research, allowing us to directly compare cortical thinning patterns between DSAD and ADAD.^9^

## 2 METHODS

### 2.1 Participants

Data from adult (≥25 years old) participants with DS in the first session of the ongoing multisite longitudinal study, Alzheimer's Biomarker Consortium‐Down Syndrome (ABC‐DS),^20^ were used for cross‐sectional analyses. Genetic testing was conducted when karyotype was unknown. Apolipoprotein E (APOE) ε4 status was assessed using a KASP genotyping assay (LGC Genomics; Beverly, MA, USA). Individuals with at least one ε4 allele were considered ε4 carriers. All participants and/or their legally authorized representatives provided informed assent or consent before participating. A common study protocol was used to obtain approval from the Institutional Review Boards at each site that conforms with the ethical standards of the 1964 Declaration of Helsinki and its amendments. ADAD participant recruitment has been previously described.^9^

### 2.2 Imaging acquisition and processing

#### 2.2.1 MRI imaging

High‐resolution T1‐MPRAGE scans were obtained using 3T MRI scanners and processed with FreeSurfer 5.3^21^ to generate vertex‐wise maps of cortical thickness and subcortical volume. All FreeSurfer output used in these analyses passed the same quality control criteria as previous ADAD research.^9^ Cortical thickness maps were smoothed with a full‐width half maximum of 10 mm and resampled to fsaverage space for consistency with previous ADAD research.^9^

#### 2.2.2 Amyloid imaging

Participants underwent amyloid positron emission tomography (PET) scans using Pittsburgh compound B (PiB) (*n* = 115) or florbetapir (*n* = 63) as the radiotracer. Scans were registered to FreeSurfer and corrected for partial volume effects. Standard uptake value ratio was calculated using the cerebellar gray matter as the reference. Key regions ^22^ were averaged and transformed into centiloids.^23^ Participants with centiloids ≥ 16.4 for PiB or ≥ 20.6 for florbetapir were classified as Aβ positive,^24^ matching previous work in ADAD^9^ and consistent with other research in DS.^25^ For more information, see Supplemental Methods.

### 2.3 Cognition

Clinical AD status was determined by consensus conferences in accordance with the recommendations of the AAMR‐IASSID working group for the establishment of criteria for the diagnosis of dementia in individuals with developmental disability.^26^ Consensus was based on caregiver interviews, medical, clinical, and cognitive testing considered in reference to baseline IQ and any recent major life transitions or events and blinded to imaging data.^20^ Participants were categorized as cognitively stable (CS), mild cognitive impairment in DS (MCI‐DS), dementia (DEM), or unable to determine (excluded). The MCI‐DS and DEM groups were combined into an impaired (IMP) group to match analyses conducted in ADAD.^9^

### 2.4 Statistical analyses

#### 2.4.1 FreeSurfer cortical thickness analyses

Cortical thickness analyses mirrored those performed in ADAD.^9^ Briefly, cortical thickness in cognitively stable amyloid negative (CS−), cognitively stable amyloid positive (CS+), and impaired amyloid positive (IMP+) groups were compared at the vertex level using FreeSurfer's mri_glmfit command, with sex and age as covariates. This diverges from previous ADAD research which only compared CS− and IMP+.^9^ Group differences were tested at multiple vertex‐wise *p*‐values and were cluster corrected (*p ≤* 0.001). The resulting cluster maps were considered potential cortical signatures. The average thickness in each cortical signatures was determined for each individual and tested using receiver operating characteristic (ROC) analyses to identify which signature(s) best differentiated groups. The map with the best area under the curve (AUC) for each comparison was deemed the ideal cortical signature for differentiating these groups. The effect size of group thickness differences was also calculated. This procedure was performed for each cortical signature for each group comparison (CS− vs CS+, CS+ vs IMP+, and CS− vs IMP+), even if a vertex‐wise group difference was not observed as the combined vertex and cluster significance correction may miss small effects. Significance was determined using independent sample *t*‐tests and a false discovery rate multiple comparison correction for every cortical signature. A similar ROC and effect size approach was applied to subcortical gray matter volumes corrected for intracranial volume.^27^ See Supplemental Methods for more information and Supplemental Figure 1 for a graphic representation of this analytic approach.

**RESEARCH IN CONTEXT**

1. **Systematic review**: The authors reviewed the literature using PubMed. While previous research has identified structural neuroimaging biomarkers for Down syndrome Alzheimer's disease (DSAD), these studies have not evaluated structural differences as a staging tool.
2. **Interpretation**: Our findings add a new method of staging Alzheimer's disease (AD) pathology in Down syndrome (DS). DSAD has a greater effect on cortical thickness relative to subcortical volume at later disease stages. DSAD progression is relatively uniform, more extensive, and more severe compared to ADAD.
3. **Future directions**: This manuscript only explores a relatively narrow set of structural imaging biomarker, future work may examine other modalities. AD drug trials in DS may use the brain regions and thresholds we have identified for evaluating future treatment efficacy.

#### 2.4.2 Comparison to ADAD

Our image processing, quality control, and analyses were nearly identical to previously published work in ADAD^9^, as adopting the same approach allows for comparison of thinning patterns between ADAD and DSAD. The only difference was that a subset of CS− participants from the FreeSurfer analyses were withheld to avoid biasing ROC analyses. We compared CS−, CS+, and IMP+ while the previous analyses only compared CS− to IMP+.

To assess the similarity between genetic forms of early‐onset Alzheimer's dementia, we compared the DSAD and ADAD specific CS− vs IMP+ effect size maps. Individual participant data were unavailable for ADAD, making direct comparison of ADAD and DSAD participants impossible. Additionally, other AD group differences (e.g., CS− vs CS+) were not measured in the ADAD cohort. The ADAD effect size maps were featured in previously published research^9^ with the relevant FreeSurfer surface files provided to us. The DSAD effect size maps were automatically generated as part of the FreeSurfer analyses. Effect size maps were used instead of cortical signatures due to their robustness to sample size variations. We identified regions with ≥ 0.2 mm thinning in each group's map and calculated the Dice coefficient^28^ to quantify the degree of overlap in thinning patterns between DSAD and ADAD. Dice coefficients range from 1 (complete overlap) to 0 (complete separation). We also evaluated spatial correlations between groups and hemispheres at the vertex level.

## 3 RESULTS

### 3.1 Participants

This study included 178 adults with DS, with 57% being male. Participants were categorized as either amyloid negative/CS− (*n* = 106), preclinical/CS+ (*n = *45), or symptomatic/IMP+ (*n = *27). Among the IMP+ group, 15 participants were MCI‐DS and 12 participants were DEM (see Table 1 for additional information). A total of 15 participants were excluded: 9 had uncertain cognitive status, 5 did not have amyloid PET data, and 1 was impaired despite being amyloid PET negative. There were no significant differences in age, centiloid values, sex, or APOE ε4 status for the 80% of the CS− sample used in the FreeSurfer analyses and the 20% withheld for the ROC analyses (Supplementary Table 1).

**TABLE 1:** Demographics.

	CS−	CS+	IMP+
Parameter	*n = *106	*n = *45	*n = *27
Sex (% male)	54 (51%)	28 (62%)	19 (70%)
Age	36.20 (6.20)	48.91 (6.18)	51.93 (4.33)
Amyloid in centiloid	1.05 (7.42)	51.96 (23.82)	76.37 (35.32)
% APOE ε4+ (*n*)	21% (22)	13% (6)	30% (8)

*Note*: Demographic information for the ABC‐DS participants. Age significantly increased in each stage of pathology (p < 0.001 for CS− to CS+ and *p = *0.018 for CS+ to IMP+) as did centiloid (*p <* 0.001 for CS− to CS+ and *p = *0.002 for CS+ to IMP+). There were no significant differences in sex or APOE e4 prevalence between groups.

Abbreviations: APOE, apolipoprotein E; CS−, cognitive stable amyloid negative; CS+, cognitively stable amyloid positive; IMP+, impaired amyloid positive.

### 3.2 Cortical signatures and thickness differences between groups

#### 3.2.1 Group differences at the vertex and cluster level

Cluster corrected vertex‐wise analyses did not identify any significant difference between CS− and CS+ participants. Significantly thinner cortices were observed in primarily the parietal and temporal regions when comparing CS− to IMP+ and CS+ to IMP+, with a rightward asymmetry in thickness differences. Increasing the vertex threshold narrowed clusters to only the parietal lobe (See Supplemental Figure 1.1 and 1.2).

#### 3.2.2 Cortical signatures

**FIGURE 1:**
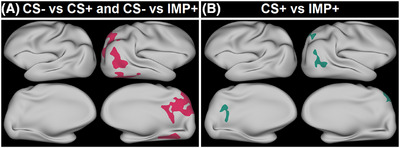
Ideal cortical signatures. The regions where cortical thickness best differentiates groups when comparing (A) CS− vs CS+ and CS− vs IMP+ and (B) CS+ vs IMP+. CS−, cognitively stable amyloid negative; CS+, cognitively stable amyloid positive; IMP+, impaired amyloid positive.

The ideal cortical signatures (detailed in the Supplemental Results) had thinner cortices in relatively diffuse clusters in the right hemisphere of the parietal and temporal lobes (CS− vs IMP+, p ≤ 0.005). These signatures best differentiated CS− from CS+ (AUC = 0.830) and CS− from IMP+ (AUC = 0.960). Thinner cortices in more focal clusters in the left precuneus and right lateral parietal lobes (CS− vs IMP+, *p* ≤ 0.0005) best differentiated CS+ from IMP+ (AUC = 0.805) (Figure 1). These cortical signatures can be accessed online (https://github.com/JTKennedyWUSTL/DS‐Cortical‐Signature).

**FIGURE 2:**
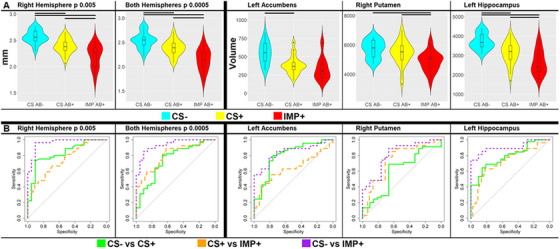
Group distributions of thickness in cortical signatures and subcortical volume. Cortical signatures and subcortical regions that best differentiate stage of AD in adults with DS. CS−, CS+, IMP+. (A) Violin plots showing distribution of cortical thickness in cortical signatures (CS− vs IMP+) and subcortical volumes. Cortical signatures and hippocampal volume decrease with increasing AD severity, striatal volumes show only early or late volume differences. Significant group differences are indicated with a black bar at the top of the figure. (B) ROC curves showing the ability of cortical signatures and subcortical volumes to differentiate groups, with greater AUC indicating better group differentiation. The cortical signatures are from the CS− vs IMP+ analyses that best differentiate AD stages. CS− vs IMP+ differentiation outperformed other comparisons, CS− vs CS+ was best identified by diffuse thinning in the right hemisphere and left accumbens, and CS + vs IMP + was best differentiated using focal bilateral thinning and right putamen volume. AD, Alzheimer's disease; AUC, area under the curve; CS−, cognitively stable amyloid negative; CS+, cognitively stable amyloid positive; DS, Down syndrome; IMP+, impaired amyloid positive; ROC, receiver operating characteristic.

Thickness differences were compared across the AD continuum (CS− vs CS+, CS+ vs IMP+, and CS− vs IMP+) using cortical signatures rather than vertex data (see Supplemental Table 2 and Figure 2A). Overall, thinner cortices were observed with increasing disease severity. Although not statistically significant for vertex/cluster‐level analyses, cortical signatures were able to significantly differentiate CS− from CS+, even when the cortical signatures were derived from the comparison of other groups. The unthresholded effect size maps for each group comparison are shown in Supplemental Figure 1.1. While the vertex/cluster level were not significantly different, these maps indicate that thinning during the later stages of AD pathology are present in parietal regions even at earlier stages (CS− vs CS+). The most significant reduction in cortical signature thickness was observed between CS+ and IMP+. See the Supplemental Results for spatial correlation findings.

### 3.3 Subcortical volume differences across AD pathology

Subcortical volumes were compared among different stages of AD pathology. Significant differences were found for several brain regions (e.g., CS− to CS+ and CS+ to IMP+). CS+ individuals had smaller bilateral accumbens and hippocampi and left amygdala and putamen compared to CS− individuals. IMP+ individuals had smaller bilateral hippocampi, putamen, and right amygdala relative to CS+ (Figure 2A). The volume of the hippocampus and left putamen differed across all stages of AD pathology.

Among subcortical regions, the left accumbens was the most effective in differentiating CS− from CS+ (0.801), while the right putamen was most effective in distinguishing CS+ from IMP+ (0.739). This suggests that specific subcortical regions were more affected during different stages of AD pathology. When comparing CS− and IMP+, hippocampal volume best differentiated groups (AUC* = *0.905; Figure 2B).

### 3.4 Cortical signature maps vs subcortical volumes

The differences between CS+ and IMP+ were greater for cortical signature maps compared to subcortical regions (Cohen's *d* = 1.199 for cortical maps, 0.754 for subcortical regions, *t* = 8.473). Effect sizes were similar in cortical and subcortical measures for CS− compared to CS+ (Cohen's *d* = 1.003 for cortical maps, 1.000 for subcortical regions, *t = *0.040) (Supplementary Table 2). Overall, AUCs for cortical signatures were slightly better than subcortical regions (averaging 0.865 for cortical vs 0.815 for subcortical). However, there were no significant differences in the best AUCs between cortical and subcortical regions (*p = *0.71 for CS− vs CS+, *p = *0.29 for CS+ vs IMP+, and *p = *0.25 for CS− vs IMP+) (Supplementary Table 2).

### 3.5 DSAD and ADAD overlap

**FIGURE 3:**
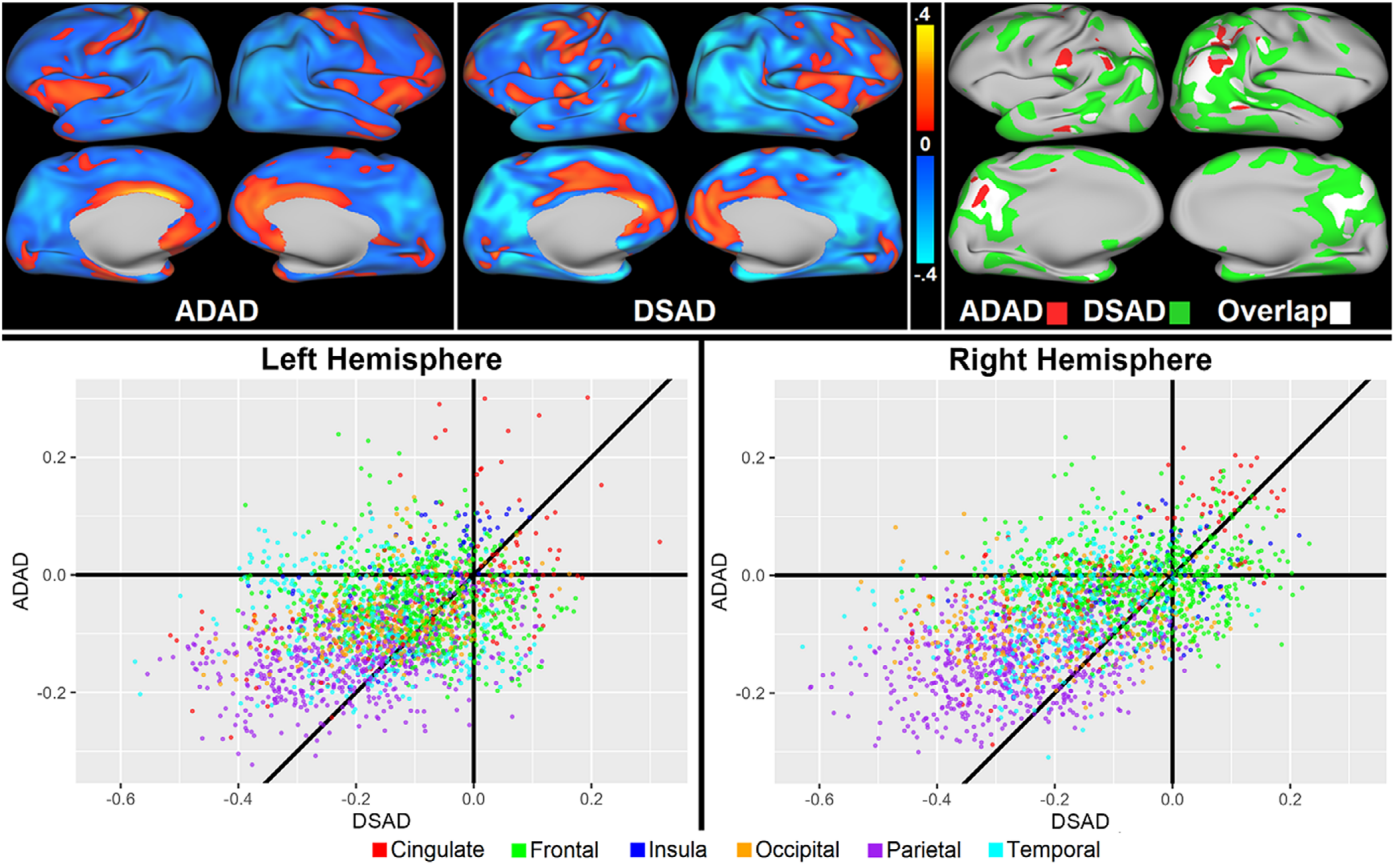
Effect and group overlap maps. Top: Millimeters of difference in cortical thickness between CS− and IMP+ individuals with AD (left) and DS (middle) AD. Thinning with increasing AD stage is shown in blue, increased thinning a lighter blue; thickening shown in red, increased thickening in yellow. The overlap between thinning patterns (at ≥ 0.2 mm threshold) shown on the right, green clusters indicate where thinning is seen in DS, red clusters indicate thinning seen in ADAD, white clusters indicate thinning in both groups. Both groups overlap primarily in the parietal with DS thinning more diffuse. Bottom: Scatterplot showing thickness differences at corresponding points in the cortex for DSAD and ADAD (downsampled to 2,562 vertices from 163,842). Points above the identity line and below 0 on the *y*‐axis show negative thickness differences when comparing CS− to IMP+ participants tend to be greater in DSAD relative to ADAD, with differences primarily in parietal and temporal regions. AD, Alzheimer's disease; ADAD, autosomal‐dominant Alzheimer's disease; AUC, area under the curve; CS−, cognitively stable amyloid negative; CS+, cognitively stable amyloid positive; DS, Down syndrome; IMP+, impaired amyloid positive.

When comparing effect size maps for cortical thickness differences between DSAD and ADAD, the lateral and medial parietal lobes were the most commonly affected regions (Figure 3, top). However, there was a low degree of overlap (Dice values of 0.196 for the left and 0.233 for the right hemisphere) due to the more widespread effects observed in DSAD compared to ADAD (Figure 3, bottom, and Supplemental Table 3). While DSAD and ADAD patterns were similar (*Rho = *0.429), observed changes with ADAD were more symmetrical. Spatial correlations for the various groups are shown in Supplemental Results. Finally, the cortical signatures seen with DSAD were better in differentiating amyloid positivity compared to ADAD (0.772 in the left and 0.830 in the right hemisphere for DSAD vs 0.547 and 0.540 for ADAD).

## 4 DISCUSSION

We observed differences in cortical thickness in the posterior parietal and temporal cortices, as well as reduced volumes in subcortical structures, with increasing DSAD stage. Contrary to our hypotheses, cortical thickness was a better indicator of AD stage than subcortical volume, and the striatum was better at group differentiation than the hippocampus. Cortical thickness difference patterns were similar for DSAD and ADAD, although the spatial extent and magnitude differences among groups were greater in DSAD. The cortical signature for DSAD was more effective in differentiating groups based on amyloid positivity (AUC* = *0.83) compared to previously published values for ADAD (0.54) or LOAD (0.65),^9^ suggesting a more consistent and predictable course.

Cortical thickness patterns differentiated DSAD across disease stages, although the specific regions that best distinguished the groups varied depending on the AD stage. Amyloid accumulation in CS individuals was associated with lower cortical thickness in the right parietal and fusiform cortices. This could be identified using cortical signatures derived from comparing thickness across all stages of pathology, but not with vertex/cluster‐level analyses comparing CS amyloid‐negative and amyloid‐positive participants. Standard approaches to identifying differences in cortical thickness may be too restrictive at the early stages of AD pathology without larger sample sizes. The cortical signatures identified in our analyses may represent a more sensitive approach to identifying early AD pathology. When comparing CS+ and IMP+, focal differences were seen in the left precuneus and right lateral parietal cortex. These results suggest that, while amyloid is associated with widespread structural differences, thinner parietal cortices specifically may be associated with cognitive impairment.

Cortical thickness outperformed subcortical volume in differentiating groups. Although effects were not observed at the vertex level for CS− versus CS+ for most analyses,^29^ cortical signatures were able to detect significant effects. This finding suggests that early effects are present but have not yet reached the vertex and cluster thresholds required for significance in a vertex‐based analysis. The thickness differences observed with amyloid accumulation are similar to the patterns observed when comparing CS− versus IMP+, particularly in the medial parietal cortex. These results suggest that the cortical thickness differences that eventually lead to cognitive impairment in DSAD occur early in the disease but have not yet progressed to the threshold required to cause noticeable impairment.

In DSAD, structural differences in subcortical regions did not follow the expected pattern of differences across stages observed in LOAD. The best predictors of AD stage were subcortical structures besides the hippocampus. This observation was interesting considering that hippocampal volume loss is a classical signature of LOAD pathology.^18^ In LOAD, other subcortical structures demonstrate volumetric decline, although typically at later stages of impairment after hippocampal volume has already decreased.^16^ The presence of amyloid plaques in striatal structures early in DSAD but not in LOAD^30^ may relate to the atypical volume differences observed in DS. These results highlight how genetic (DSAD and ADAD) and LOAD pathology may exhibit different patterns of structural change, necessitating the use of group‐specific measures for neurodegeneration.

Interestingly, the subcortical regions that best differentiated stages of AD pathology were inconsistent. The accumbens best differentiated CS− from CS+ but performed poorly for discriminating CS+ from IMP+. Conversely, the putamen had poor discriminative validity for distinguishing amyloid positivity but performed better in identifying symptomatic DSAD. The caudate performed poorly in differentiating all stages. These results suggest not only atypical striatal involvement in DSAD but also non‐uniformity. Future studies that will develop a standardized “signature” for neurodegenerative changes in DSAD should take this into account.

When comparing the spatial pattern of cortical differences in DSAD and ADAD, we observed a high degree of overlap. Group effects were strongest in the parietal lobe for both groups. Although spatial patterns were similar, the area of thickness differences in DSAD was six times greater than ADAD. It is worth noting that, while 74% of the affected areas in ADAD were also implicated in DSAD, unique patterns of thinner cortices were observed in DSAD. ADAD effects were generally bilateral, while effects in DSAD were more lateralized. The right hemisphere was more affected in DSAD and differentiated AD stages better than the left. Furthermore, unlike DSAD, ADAD has relatively preserved medial temporal lobes, a classical region of AD pathology in LOAD.^7^, ^8^, ^9^ The pattern of cortical effects is similar to the pattern of tau accumulation in DSAD and ADAD. The greater differences in DSAD may reflect that tau levels are significantly higher for a given amount of amyloid in DSAD,^31^ leading to greater neurodegeneration.

A key disparity for cortical differences between DSAD and ADAD was its suitability as a biomarker used for disease staging. Previous ADAD research examined how well the cortical signature differentiated CS− from CS+, resulting in an AUC slightly better than chance at 0.54, compared to DSAD's 0.83. This may reflect a more uniform presentation in DSAD due to the pathology arising from a common genetic effect. In contrast, ADAD comprises individuals with mutations in three different genes, with the location of the mutation along these genes influencing the AD pathology.^32^ This likely introduces heterogeneity in structural changes as ADAD progresses, limiting the applicability of a common cortical signature. Similarly, the low AUC in the LOAD analyses (0.65) may reflect that LOAD pathology is linked to several risk factors,^33^, ^34^ and isolating differences based on a common assumed etiology, such as APOE ε4 homozygotes, may yield a more reliable cortical signature.

### 4.1 Limitations and future directions

There are several limitations to the current study. We were limited by the number of amyloid positive, cognitively impaired individuals with DS. The lack of a subset specifically withheld for the ROC analyses may have inflated AUCs resulting from comparisons involving this group. Future studies that include more impaired individuals are needed. We were unable to make direct comparisons to the ADAD cohort due to only having access to effect size maps. As we used sample‐size independent effect size maps to compare the groups, some regions highlighted when thresholding the maps were non‐significant in the group difference analyses. Additionally, our findings were based on cross‐sectional analyses, meaning they were derived from group discrepancies rather than individual changes as AD progresses and may incorporate unaccounted individual differences. The longitudinal nature of ABC‐DS mitigates this limitation in future work. With forthcoming longitudinal imaging data, we may be able to identify individuals who convert to amyloid‐positive or symptomatic between waves to more concretely identify the regions affected by AD pathology while minimizing individual differences.

## 5 CONCLUSIONS

A distinct pattern of cortical thickness differences was identified in individuals with DS that can effectively identify preclinical and symptomatic AD. DSAD is characterized by posterior effects that share similarities with ADAD but are greater in magnitude and spatial extent. Compared to LOAD or ADAD, DSAD cortical signatures demonstrate superior discriminative validity. While subcortical volumes can stage AD pathology, they are not as effective as cortical signatures. By deciphering brain structural signatures, we hope to gain valuable insights into the progression of DSAD, enhancing our understanding of the disease and potentially facilitating early interventions.

## CONFLICT OF INTEREST STATEMENT

The authors declare no conflicts of interest. Author disclosures are available in the Supporting Information.

## CONSENT STATEMENT

All participants and their legally authorized caregivers provided informed assent or consent.

## Supporting information



Supporting information

Supporting information

Supporting information

Supporting information

Supporting information

Supporting information

## Appendix

ABC‐DS and DIAN collaborators are indexed online at https://www.nia.nih.gov/research/abc‐ds#sites and https://dian.wustl.edu/our‐research/observational‐study/dian‐observational‐study‐sites/.

## References

[1] Jack CR , Bennett DA , Blennow K , et al. A/T/N: an unbiased descriptive classification scheme for Alzheimer disease biomarkers. Neurology. 2016;87(5):539‐547. doi:10.1212/WNL.0000000000002923 27371494 PMC4970664

[2] Bateman RJ , Aisen PS , De Strooper B , et al. Autosomal‐dominant Alzheimer's disease: a review and proposal for the prevention of Alzheimer's disease. Alzheimers Res Ther. 2011;3(1):1. doi:10.1186/alzrt59 21211070 PMC3109410

[3] Fortea J , Zaman SH , Hartley S , Rafii MS , Head E , Carmona‐Iragui M . Down syndrome‐associated Alzheimer's disease: a genetic form of dementia. Lancet Neurol. 2021;20(11):930‐942. doi:10.1016/S1474-4422(21)00245-3 34687637 PMC9387748

[4] Bateman RJ , Xiong C , Benzinger TL , et al. Clinical and biomarker changes in dominantly inherited Alzheimer's Disease. N Engl J Med. 2012;367(9):795‐804. doi:10.1056/NEJMoa1202753 22784036 PMC3474597

[5] Boerwinkle AH , Gordon BA , Wisch J , et al. Comparison of amyloid burden in individuals with Down syndrome versus autosomal dominant Alzheimer's disease: a cross‐sectional study. Lancet Neurol. 2023;22(1):55‐65. doi:10.1016/S1474-4422(22)00408-2 36517172 PMC9979840

[6] Rafii MS , Fortea J . Down Syndrome in a new era for Alzheimer Disease. JAMA. 2023;330(22):2157‐2158. doi:10.1001/jama.2023.22924 37991807 PMC11324235

[7] Dickerson BC , Bakkour A , Salat DH , et al. The cortical signature of Alzheimer's Disease: regionally specific cortical thinning relates to symptom severity in very mild to mild ad dementia and is detectable in asymptomatic amyloid‐positive individuals. Cereb Cortex N Y NY. 2009;19(3):497‐510. doi:10.1093/cercor/bhn113 PMC2638813 18632739

[8] Wang L , Benzinger TL , Hassenstab J , et al. Spatially distinct atrophy is linked to β‐amyloid and tau in preclinical Alzheimer disease. Neurology. 2015;84(12):1254‐1260. doi:10.1212/WNL.0000000000001401 25716355 PMC4366088

[9] Dincer A , Gordon BA , Hari‐Raj A , et al. Comparing cortical signatures of atrophy between late‐onset and autosomal dominant Alzheimer disease. NeuroImage Clin. 2020;28:102491. doi:10.1016/j.nicl.2020.102491 33395982 PMC7689410

[10] Fortea J , Vilaplana E , Carmona‐Iragui M , et al. Clinical and biomarker changes of Alzheimer's disease in adults with Down syndrome: a cross‐sectional study. Lancet Lond Engl. 2020;395(10242):1988‐1997. doi:10.1016/S0140-6736(20)30689-9 PMC7322523 32593336

[11] Benejam B , Aranha MR , Videla L , et al. Neural correlates of episodic memory in adults with Down syndrome and Alzheimer's disease. Alzheimers Res Ther. 2022;14(1):123. doi:10.1186/s13195-022-01064-x 36057615 PMC9440567

[12] Annus T , Wilson LR , Acosta‐Cabronero J , et al. The Down syndrome brain in the presence and absence of fibrillar β‐amyloidosis. Neurobiol Aging. 2017;53:11‐19. doi:10.1016/j.neurobiolaging.2017.01.009 28192686 PMC5391869

[13] Padilla C , Montal V , Walpert MJ , et al. Cortical atrophy and amyloid and tau deposition in Down syndrome: a longitudinal study. Alzheimers Dement Diagn Assess Dis Monit. 2022;14(1):e12288. doi:10.1002/dad2.12288 PMC8974205 35386472

[14] Lao PJ , Handen BL , Betthauser TJ , et al. Longitudinal changes in amyloid positron emission tomography and volumetric magnetic resonance imaging in the nondemented Down syndrome population. Alzheimers Dement Amst Neth. 2017;9:1‐9. doi:10.1016/j.dadm.2017.05.001 PMC5454131 28603769

[15] Benzinger TLS , Blazey T , Jack CR , et al. Regional variability of imaging biomarkers in autosomal dominant Alzheimer's disease. Proc Natl Acad Sci USA. 2013;110(47):E4502‐4509. doi:10.1073/pnas.1317918110 24194552 PMC3839740

[16] Roh JH , Qiu A , Seo SW , et al. Volume reduction in subcortical regions according to severity of Alzheimer's disease. J Neurol. 2011;258(6):1013‐1020. doi:10.1007/s00415-010-5872-1 21240517

[17] Feng F , Huang W , Meng Q , et al. Altered volume and structural connectivity of the hippocampus in Alzheimer's disease and amnestic mild cognitive impairment. Front Aging Neurosci. 2021;13:705030. doi:10.3389/fnagi.2021.705030 34675796 PMC8524052

[18] Schuff N , Woerner N , Boreta L , et al. MRI of hippocampal volume loss in early Alzheimer's disease in relation to ApoE genotype and biomarkers. Brain. 2009;132(4):1067‐1077. doi:10.1093/brain/awp007 19251758 PMC2668943

[19] Koenig KA , Oh SH , Stasko MR , et al. High resolution structural and functional MRI of the hippocampus in young adults with Down syndrome. Brain Commun. 2021;3(2):fcab088. doi:10.1093/braincomms/fcab088 33977271 PMC8100000

[20] Handen BL , Lott IT , Christian BT , et al. The Alzheimer's Biomarker Consortium‐Down Syndrome: rationale and methodology. Alzheimers Dement (Amst). 2020;12(1):e12065. doi:10.1002/dad2.12065 32775597 PMC7396809

[21] Dale AM , Fischl B , Sereno MI . Cortical surface‐based analysis. I. Segmentation and surface reconstruction. Neuroimage. 1999;9(2):179‐194. doi:10.1006/nimg.1998.0395 9931268

[22] Su Y , D'Angelo GM , Vlassenko AG , et al. Quantitative analysis of PiB‐PET with FreeSurfer ROIs. PLoS One. 2013;8(11):e73377. doi:10.1371/journal.pone.0073377 24223109 PMC3819320

[23] Su Y , Flores S , Hornbeck RC , et al. Utilizing the centiloid scale in cross‐sectional and longitudinal pib pet studies. NeuroImage Clin. 2018;19:406‐416. doi:10.1016/j.nicl.2018.04.022 30035025 PMC6051499

[24] Klunk WE , Koeppe RA , Price JC , et al. The Centiloid Project: standardizing Quantitative Amyloid Plaque Estimation by PET. Alzheimers Dement. 2015;11(1):1‐15.e4. doi:10.1016/j.jalz.2014.07.003 25443857 PMC4300247

[25] Zammit MD , Tudorascu DL , Laymon CM , et al. PET measurement of longitudinal amyloid load identifies the earliest stages of amyloid‐beta accumulation during Alzheimer's disease progression in Down syndrome. Neuroimage. 2021;228:117728. doi:10.1016/j.neuroimage.2021.117728 33421595 PMC7953340

[26] Janicki MP , Heller T , Seltzer GB , et al. Practice guidelines for the clinical assessment and care management of Alzheimer's disease and other dementias among adults with intellectual disability. AAMR‐IASSID Workgroup on Practice Guidelines for Care Management of Alzheimer's Disease among Adults with Intellectual Disability. J Intellect Disabil Res. 1996;40(Pt 4):374‐382. 8884593

[27] Buckner RL , Head D , Parker J , et al. A unified approach for morphometric and functional data analysis in young, old, and demented adults using automated atlas‐based head size normalization: reliability and validation against manual measurement of total intracranial volume. Neuroimage. 2004;23(2):724‐738. doi:10.1016/j.neuroimage.2004.06.018 15488422

[28] Dice LR . Measures of the Amount of Ecologic Association Between Species. Ecology. 1945;26(3):297‐302. doi:10.2307/1932409

[29] Mak E , Bickerton A , Padilla C , et al. Longitudinal trajectories of amyloid deposition, cortical thickness, and tau in Down syndrome: a deep‐phenotyping case report. Alzheimers Dement (Amst). 2019;11:654‐658. doi:10.1016/j.dadm.2019.04.006 31909173 PMC6939035

[30] Cohen AD , McDade E , Christian B , et al. Early striatal amyloid deposition distinguishes Down syndrome and autosomal dominant Alzheimer's disease from late‐onset amyloid deposition. Alzheimers Dement. 2018;14(6):743‐750. doi:10.1016/j.jalz.2018.01.002 29477284 PMC5994364

[31] Wisch JK , McKay NS , Boerwinkle AH , et al. Comparison of tau spread in people with Down syndrome versus autosomal‐dominant Alzheimer's disease: a cross‐sectional study. Lancet Neurol. 2024;23(5):500‐510. doi:10.1016/S1474-4422(24)00084-X 38631766 PMC11209765

[32] Joseph‐Mathurin N , Feldman RL , Lu R , et al. Presenilin‐1 mutation position influences amyloidosis, small vessel disease, and dementia with disease stage. Alzheimers Dement J Alzheimers Assoc. 2024;20(4):2680‐2697. doi:10.1002/alz.13729 PMC11032566 38380882

[33] Elias‐Sonnenschein LS , Bertram L , Visser PJ . Relationship between genetic risk factors and markers for Alzheimer's Disease pathology. Biomark Med. 2012;6(4):477‐495. doi:10.2217/bmm.12.56 22917148

[34] Zhang XX , Tian Y , Wang ZT , Ma YH , Tan L , Yu JT . The epidemiology of Alzheimer's Disease modifiable risk factors and prevention. J Prev Alzheimers Dis. 2021;8(3):313‐321. doi:10.14283/jpad.2021.15 34101789

