# Supplementary material for: Decoding brain structure to stage Alzheimer's disease pathology in Down syndrome
Source: Alzheimers Dement. 2025 Jan 14;21(2):e14519. doi: 10.1002/alz.14519 (PMC11848172; doi:10.1002/alz.14519)
Supplement: Supplementary file 1 — Supporting information [file ALZ-21-e14519-s006.docx]

**Supplementary Table 1: Demographics**

|  | ABC-DS | | | | | DIAN | | |
| --- | --- | --- | --- | --- | --- | --- | --- | --- |
|  | CS- | CS- 80% | CS- 20% | CS+ | IMP+ | CS- | CS+ | IMP+ |
|  | *n =* 106 | *n* = 85 | *n* = 21 | *n* = 45 | *n* = 27 | *n* =145 | *n* = 76 | *n* = 48 |
| Gender (% male) | 54 (51%) | 44 (52%) | 10 (48%) | 28 (62%) | 19 (70%) | 58 (40.0) | 37 (48.7) | 18 (37.5) |
| Age | 36.2 (6.2) | 36.1 (6.4) | 36.4 (5.7) | 48.9 (6.2) | 51.9 (4.3) | 38.5 (11.3) | 37.2 (9.0) | 44.8 (10.6) |
| SUVR (PIB) | 1 (0.1) | 1 (0.1) | 1 (0.1) | 2.1 (0.6) | 3 (0.8) | 1.05 (0.07) | 2.05 (0.61) | 2.89 (1.08) |
| SUVR (AV45) | 1.1 (0.1) | 1.1 (0.1) | 1 (0.1) | 1.8 (0.4) | 2.1 (0.7) | - | - | - |
| Amyloid in Centiloids | 1.1 (7.4) | 1.1 (7.6) | 0.9 (6.9) | 52 (23.8) | 76.4 (35.3) | -0.4 (3.1) | 44.8 (27.5) | 82.5 (48.6) |
| *n* APOE e4+ (%) | 22 (21%) | 18 (21%) | 4 (19%) | 6 (13%) | 8 (30%) | 42 (29%) | 26 (34%) | 15 (31%) |
| APP/PSEN1/2 | - | - | - | - | - | 30/97/18 | 6/59/11 | 7/39/2 |

Full demographic information for the ABC-DS and DIAN participants. CS-: Cognitive stable amyloid negative, 80%: 80% of CS- used in vertex-wise analyses, 20%: 20% of CS- used for ROC analyses, CS+: Cognitively stable amyloid positive, IMP+: Impaired amyloid positive. SUVR: Standardized uptake value ratio. APP/PSEN1/2: Number of participants with a mutation in the APP, PSEN1, or PSEN2 genes.
