# Supplementary material for: Decoding brain structure to stage Alzheimer's disease pathology in Down syndrome
Source: Alzheimers Dement. 2025 Jan 14;21(2):e14519. doi: 10.1002/alz.14519 (PMC11848172; doi:10.1002/alz.14519)
Supplement: Supplementary file 2 — Supporting information [file ALZ-21-e14519-s003.docx]

**Supplementary Table 2: Group differences and discriminative ability of cortical signatures and subcortical volumes**

|  | Signature/Region | CS- vs CS+ | | | | | CS+ vs IMP+ | | | | | CS- vs IMP+ | | | | |
| --- | --- | --- | --- | --- | --- | --- | --- | --- | --- | --- | --- | --- | --- | --- | --- | --- |
|  |  | *d* | Thresh | Spec | Sens | *AUC* | *d* | Thresh | Spec | Sens | *AUC* | *d* | Thresh | Spec | Sens | *AUC* |
| CS- vs IMP+ | Combined 0.05 | **1.199** | 2.492 | 0.905 | 0.622 | 0.800 | **1.090** | 2.390 | 0.689 | 0.704 | 0.756 | **1.848** | 2.485 | 0.905 | 0.926 | 0.929 |
|  | Combined 0.01 | **1.278** | 2.485 | 0.905 | 0.711 | 0.814 | **1.136** | 2.400 | 0.578 | 0.815 | 0.756 | **1.946** | 2.472 | 0.905 | 0.926 | 0.942 |
|  | Combined 0.005 | **1.284** | 2.474 | 0.905 | 0.711 | 0.823 | **1.109** | 2.396 | 0.533 | 0.852 | 0.753 | **1.982** | 2.459 | 0.905 | 0.926 | 0.949 |
|  | Combined 0.001 | **1.156** | 2.463 | 0.905 | 0.667 | 0.807 | **1.124** | 2.340 | 0.733 | 0.704 | 0.762 | **2.065** | 2.457 | 0.905 | 0.963 | 0.954 |
|  | Combined 0.0005 | **0.945** | 2.494 | 0.667 | 0.800 | 0.749 | **1.294** | **2.362** | **0.622** | **0.889** | **0.805** | **1.935** | 2.338 | 0.905 | 0.852 | 0.935 |
|  | Left 0.05 | **1.037** | 2.514 | 0.905 | 0.578 | 0.772 | **0.950** | 2.501 | 0.467 | 0.926 | 0.741 | **1.667** | 2.511 | 0.905 | 0.926 | 0.907 |
|  | Left 0.01 | **0.942** | 2.420 | 0.952 | 0.511 | 0.734 | **1.008** | 2.271 | 0.889 | 0.556 | 0.750 | **1.634** | 2.431 | 0.952 | 0.815 | 0.908 |
|  | Left 0.005 | **0.895** | 2.439 | 0.905 | 0.533 | 0.731 | **1.026** | 2.312 | 0.800 | 0.593 | 0.744 | **1.645** | 2.412 | 0.952 | 0.778 | 0.896 |
|  | Left 0.001 | **0.692** | 2.764 | 0.667 | 0.711 | 0.696 | **1.053** | 2.615 | 0.644 | 0.778 | 0.770 | **1.591** | 2.711 | 0.762 | 0.926 | 0.885 |
|  | Left 0.0005 | **0.611** | 2.757 | 0.667 | 0.644 | 0.668 | **1.119** | 2.607 | 0.667 | 0.778 | 0.786 | **1.557** | 2.690 | 0.762 | 0.889 | 0.864 |
|  | Right 0.05 | **1.244** | 2.456 | 0.905 | 0.578 | 0.802 | **1.139** | 2.267 | 0.933 | 0.481 | 0.754 | **1.909** | 2.456 | 0.905 | 0.852 | 0.944 |
|  | Right 0.01 | **1.293** | 2.449 | 0.905 | 0.644 | 0.813 | **1.137** | 2.364 | 0.689 | 0.741 | 0.759 | **1.976** | 2.468 | 0.857 | 0.926 | 0.951 |
|  | Right 0.005 | **1.316** | **2.468** | **0.905** | **0.733** | **0.830** | **1.087** | 2.301 | 0.778 | 0.593 | 0.749 | **2.009** | **2.462** | **0.905** | **0.963** | **0.960** |
|  | Right 0.001 | **1.139** | 2.411 | 0.905 | 0.622 | 0.797 | **1.043** | 2.290 | 0.733 | 0.667 | 0.743 | **2.030** | 2.413 | 0.905 | 0.926 | 0.951 |
|  | Right 0.0005 | **0.887** | 2.409 | 0.667 | 0.733 | 0.729 | **1.120** | 2.228 | 0.711 | 0.741 | 0.770 | **1.794** | 2.300 | 0.810 | 0.889 | 0.905 |
|  | Right 0.0001 | 0.421 | 2.298 | 0.619 | 0.667 | 0.639 | **0.791** | 2.194 | 0.556 | 0.815 | 0.716 | **1.217** | 2.195 | 0.762 | 0.815 | 0.819 |
| CS+ vs IMP+ | Combined 0.05 | **0.960** | 2.492 | 0.857 | 0.622 | 0.748 | **1.427** | 2.393 | 0.756 | 0.778 | 0.830 | **1.928** | 2.431 | 0.905 | 0.889 | 0.935 |
|  | Combined 0.01 | **0.787** | 2.582 | 0.571 | 0.822 | 0.707 | **1.590** | **2.372** | **0.867** | **0.778** | **0.864** | **1.995** | 2.380 | 1.000 | 0.778 | 0.944 |
|  | Combined 0.005 | **0.803** | 2.639 | 0.571 | 0.889 | 0.710 | **1.542** | 2.402 | 0.822 | 0.815 | 0.858 | **2.023** | 2.452 | 0.905 | 0.889 | 0.935 |
|  | Left 0.05 | **0.835** | 2.465 | 0.762 | 0.600 | 0.712 | **1.414** | 2.395 | 0.689 | 0.889 | 0.833 | **1.857** | 2.424 | 0.905 | 0.926 | 0.929 |
|  | Left 0.01 | 0.331 | 2.692 | 0.429 | 0.822 | 0.562 | **1.177** | 2.495 | 0.756 | 0.778 | 0.813 | **1.318** | 2.497 | 0.762 | 0.778 | 0.824 |
|  | Left 0.005 | 0.331 | 2.561 | 0.476 | 0.644 | 0.443 | **1.173** | 2.528 | 0.689 | 0.926 | 0.821 | **1.415** | 2.540 | 0.714 | 0.926 | 0.854 |
|  | Right 0.05 | **0.984** | **2.491** | **0.857** | **0.578** | **0.756** | **1.364** | 2.370 | 0.822 | 0.667 | 0.808 | **1.900** | 2.484 | 0.857 | 0.889 | 0.931 |
|  | Right 0.01 | **0.853** | 2.525 | 0.714 | 0.667 | 0.724 | **1.575** | 2.354 | 0.844 | 0.741 | 0.857 | **2.032** | **2.403** | **0.952** | **0.852** | **0.950** |
|  | Right 0.005 | **0.936** | 2.510 | 0.810 | 0.644 | 0.750 | **1.498** | 2.257 | 0.956 | 0.593 | 0.831 | **1.984** | 2.496 | 0.810 | 0.926 | 0.928 |
| Subcortical | Brainstem | -0.396 | 16619 | 0.857 | 0.467 | 0.632 | 0.235 | 16779 | 0.400 | 0.815 | 0.559 | -0.129 | 15671 | 0.524 | 0.741 | 0.594 |
|  | Left Accumbens | **1.293** | **439** | **0.762** | **0.800** | **0.801** | 0.335 | 308 | 0.778 | 0.556 | 0.635 | 1.356 | 415 | 0.810 | 0.778 | 0.840 |
|  | Left Amygdala | **0.866** | 1698 | 0.619 | 0.800 | 0.733 | **0.696** | 1404 | 0.622 | 0.704 | 0.681 | **1.386** | 1419 | 0.905 | 0.704 | 0.832 |
|  | Left Caudate | 0.432 | 3611 | 0.524 | 0.711 | 0.637 | 0.149 | 3510 | 0.467 | 0.704 | 0.541 | 0.645 | 3294 | 0.857 | 0.481 | 0.677 |
|  | Left Cerebellum | 0.323 | 38912 | 0.429 | 0.756 | 0.571 | 0.367 | 32986 | 0.867 | 0.370 | 0.584 | 0.678 | 38931 | 0.429 | 0.889 | 0.658 |
|  | Left Hippocampus | **1.101** | 3363 | 0.857 | 0.644 | 0.786 | **0.832** | 2755 | 0.822 | 0.630 | 0.726 | **1.951** | **3070** | **1.000** | **0.741** | **0.905** |
|  | Left Pallidum | 0.124 | 1196 | 1.000 | 0.111 | 0.524 | 0.364 | 1416 | 0.733 | 0.519 | 0.602 | 0.564 | 1186 | 1.000 | 0.259 | 0.626 |
|  | Left Putamen | **0.817** | 5665 | 0.810 | 0.667 | 0.721 | **0.649** | 5338 | 0.622 | 0.778 | 0.693 | **1.642** | 5590 | 0.810 | 0.852 | 0.869 |
|  | Left Thalamus | -0.160 | 6944 | 0.952 | 0.267 | 0.541 | 0.080 | 6051 | 0.711 | 0.519 | 0.583 | -0.039 | 5961 | 0.905 | 0.444 | 0.586 |
|  | Left Ventral DC | 0.351 | 3734 | 0.333 | 0.889 | 0.610 | 0.043 | 3690 | 0.200 | 0.963 | 0.492 | 0.444 | 3715 | 0.333 | 0.963 | 0.623 |
|  | Right Accumbens | **1.109** | 548 | 0.667 | 0.800 | 0.780 | 0.276 | 394 | 0.733 | 0.481 | 0.585 | 1.292 | 513 | 0.762 | 0.852 | 0.834 |
|  | Right Amygdala | **0.707** | 1639 | 0.714 | 0.622 | 0.688 | **0.721** | 1575 | 0.578 | 0.778 | 0.694 | **1.469** | 1419 | 1.000 | 0.630 | 0.857 |
|  | Right Caudate | 0.191 | 3635 | 0.667 | 0.444 | 0.455 | 0.353 | 3579 | 0.489 | 0.704 | 0.584 | 0.576 | 3173 | 1.000 | 0.333 | 0.630 |
|  | Right Cerebellum | 0.496 | 38745 | 0.571 | 0.711 | 0.618 | 0.403 | 36137 | 0.622 | 0.667 | 0.629 | 0.911 | 38615 | 0.571 | 0.778 | 0.730 |
|  | Right Hippocampus | **1.106** | 3826 | 0.524 | 0.911 | 0.784 | **0.844** | 3006 | 0.733 | 0.667 | 0.719 | **1.734** | 3148 | 0.952 | 0.704 | 0.880 |
|  | Right Pallidum | 0.279 | 1356 | 0.905 | 0.400 | 0.584 | 0.477 | 1447 | 0.511 | 0.778 | 0.628 | 0.860 | 1408 | 0.762 | 0.704 | 0.741 |
|  | Right Putamen | 0.334 | 5734 | 0.667 | 0.689 | 0.605 | **0.786** | **5277** | **0.711** | **0.778** | **0.739** | **1.285** | 5610 | 0.667 | 0.889 | 0.810 |
|  | Right Thalamus | 0.364 | 6459 | 0.476 | 0.844 | 0.600 | 0.347 | 5851 | 0.622 | 0.704 | 0.627 | 0.605 | 5500 | 0.905 | 0.481 | 0.702 |
|  | Right Ventral DC | 0.386 | 3619 | 0.571 | 0.644 | 0.603 | 0.271 | 3495 | 0.489 | 0.667 | 0.556 | 0.640 | 3633 | 0.571 | 0.778 | 0.656 |

Results from Cohen's *d* effect size and receiver operating characteristic analyses comparing AD stages for cortical signatures and subcortical volumes. Cortical signatures were more likely to show significant group differences and had higher *AUC*s. Green cells with bolded values indicate significant group differences, blue cells with bolded values indicate the cortical signature/subcortical region with the best *AUC* for a set of cortical signatures/subcortical values. CS-: Cognitive stable-, CS+: Cognitively stable+, IMP+: Impaired+, *d*: effect size, Thresh: Threshold that best differentiated groups in millimeters for cortical thickness and voxels for subcortical volumes, Spec: specificity, Sens: Sensitivity, *AUC*: Area under the curve, Combined: Weighted average of both hemispheres' cortical signatures, L: Left hemisphere, R: Right hemisphere, DC: Diencephalon.
