# Supplementary material for: Decoding brain structure to stage Alzheimer's disease pathology in Down syndrome
Source: Alzheimers Dement. 2025 Jan 14;21(2):e14519. doi: 10.1002/alz.14519 (PMC11848172; doi:10.1002/alz.14519)
Supplement: Supplementary file 3 — Supporting information [file ALZ-21-e14519-s002.docx]

**Supplementary Table 3: Spatial overlap of DSAD and ADAD**

| H | Vertices | | | Dice Coeff | Overlap% | |
| --- | --- | --- | --- | --- | --- | --- |
|  | DSAD | ADAD | Overlap |  | DSAD | ADAD |
| L | 45,149 | 7,515 | 5,147 | 0.195 | 11.4 | 68.5 |
| R | 58,390 | 10,181 | 7,978 | 0.233 | 13.7 | 78.4 |
| B | 103,539 | 17,696 | 13,125 | 0.217 | 12.7 | 74.2 |

Number of vertices below a thinning threshold of -0.2 in DSAD, ADAD, and their overlap. H: Hemisphere, L: Left, R: Right, B: Both.
