# Supplementary material for: Decoding brain structure to stage Alzheimer's disease pathology in Down syndrome
Source: Alzheimers Dement. 2025 Jan 14;21(2):e14519. doi: 10.1002/alz.14519 (PMC11848172; doi:10.1002/alz.14519)
Supplement: Supplementary file 4 — Supporting information [file ALZ-21-e14519-s001.docx]

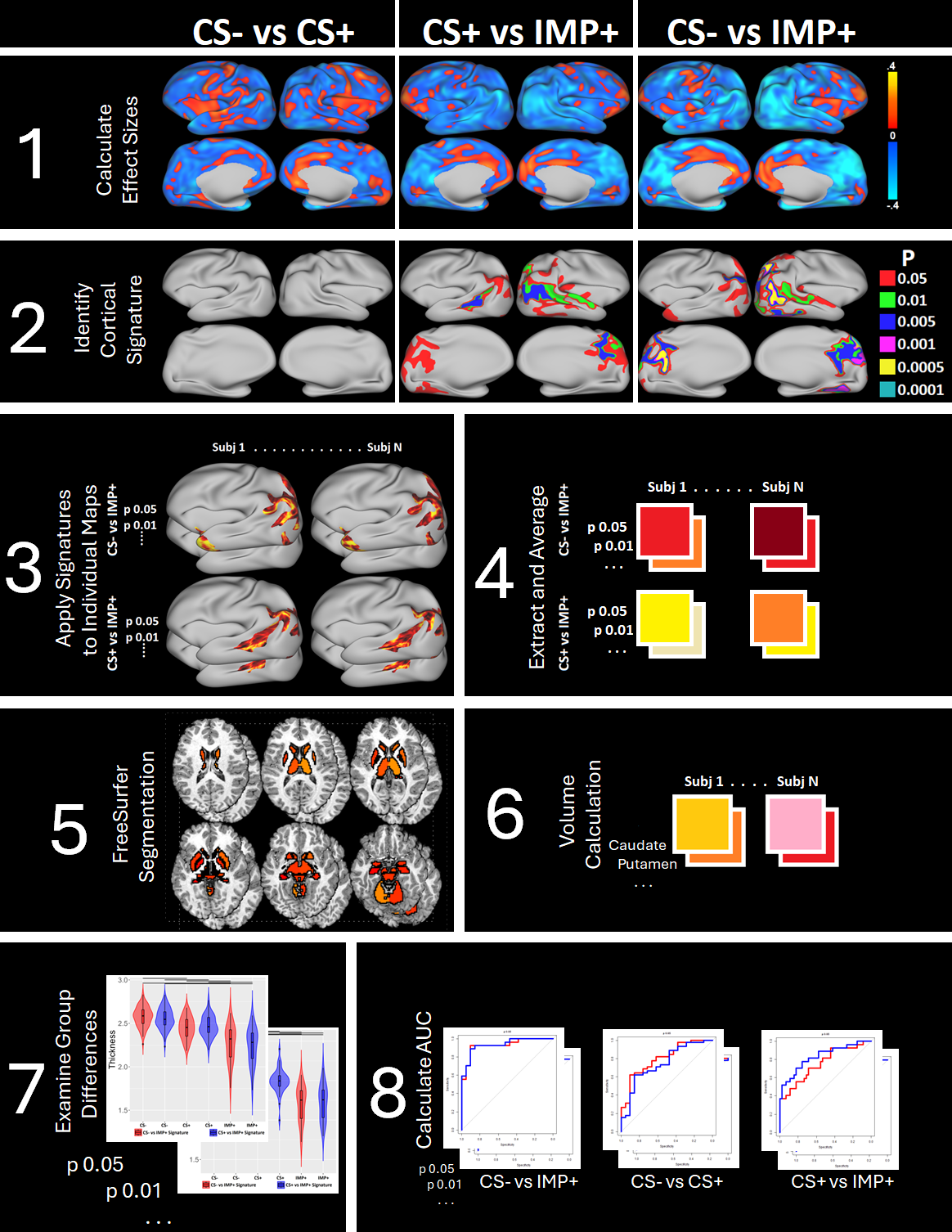


**Supplemental Figure Caption 1 Analytic Approach**

Analytic approach used to identify the ideal cortical signatures for differentiating levels of AD pathology in DS. 1) Effect size maps (differences in mm) created when calculating group differences between CS-, CS+, and IMP+. 2) Cortical signatures generated for each group comparison by thresholding group differences at varying vertexwise p-values and a 0.001 cluster wise family-wise error. 3) Every cortical signature is applied to each individual and 4) the average thickness in each cortical signature is calculated for every participant. 5) FreeSurfer identifies subcortical structures in each individual and 6) extracts regional individual volumes. 7) Cortical signature specific group differences are calculated using averaged, not vertexwise, differences. 8) Each cortical signature is evaluated for how well it can differentiate groups.
