# Supplementary material for: Decoding brain structure to stage Alzheimer's disease pathology in Down syndrome
Source: Alzheimers Dement. 2025 Jan 14;21(2):e14519. doi: 10.1002/alz.14519 (PMC11848172; doi:10.1002/alz.14519)
Supplement: Supplementary file 5 — Supporting information [file ALZ-21-e14519-s004.docx]

**1 Supplemental Methods**

**1.1 Amyloid Imaging**

Participants underwent amyloid PET scans using either 15mCi of [11C]-Pittsburgh compound B (PiB) or 10mCi of [18F]-AV45 (florbetapir) as the radiotracer. The scans commenced 50 minutes after an intravenous bolus injection and lasted for 20 minutes, divided into four five-minute frames. To analyze the PET scans, we utilized the PET Unified Pipeline,^1^ which ensured alignment with FreeSurfer segmentations. Corrections for partial volume effects were applied using a regional spread function. Standard uptake value ratio (SUVR) was using the cerebellar gray matter serving as the reference region. The mean cortical SUVR (mcSUVR) was computed by averaging the SUVR values from the following regions: precuneus, superior frontal, rostral middle frontal, lateral orbitofrontal, medial orbitofrontal, superior temporal, and middle temporal regions^1^ and transformed into centiloids to assist in cross tracer comparison.^2^ Participants with centiloids ≥ 16.4 for PiB scans or ≥ 20.6 for florbetapir scans were classified as amyloid-positive (Aβ+). Centiloids were calculated using in house methods.

**1.2 FreeSurfer Cortical Thickness Analyses**

[Insert Supplemental Figure 1]

To identify cortical regions affected by AD pathology in DS participants, a comparison was made between the impaired (IMP) amyloid positive (+) individuals, cognitively stable (CS) amyloid positive (+) individuals, and 80% of the cognitively stable amyloid negative (CS-) participants. To ensure unbiased subsequent analyses, the remaining 20% of the CS- group were withheld. However, due to the small size of the CS+ and IMP+ groups, the same withholding process was not possible. Each hemisphere was assessed separately using FreeSurfer's mri_glmfit command, with age and sex as covariates (Supplemental Figure 1.1).

To determine whether large and diffuse changes or highly significant and focal changes best differentiated the groups, thickness difference maps were thresholded at multiple vertex-wise p-values (*p* < 0.05/.01/.005/.001/.0005/.0001). This generated a set of increasingly conservative group difference maps. To eliminate small clusters that may occur by chance, a cluster size-based multiple comparison correction was performed using Monte Carlo at a *p* < 0.001 threshold across both hemispheres for each map. The surviving regions from vertex and cluster thresholding were considered as potential cortical signatures, representing regions with significant differences between groups (Supplemental Figure 1.2).

Each participant’s cortical signature specific average thickness was extracted using FreeSurfer’s mris_anatomical_stats command. An individual’s weighted average bilateral cortical signature specific thickness was calculated using the formula ((AvgThicknessL * NVerticesL + AvgThicknessR * NVerticesR) / (NVerticesL + NVerticesR)). The thickness values derived from the cortical maps were assessed in terms of their ability to differentiate groups along the AD continuum (Supplemental Figure 1.5 and 1.6). Specifically, comparisons were made between the withheld CS- (80%) group and the CS+ group, as well as between CS+ and IMP+. This yielded three sets of cortical signature differences: CS- vs IMP+, CS- vs CS+, and CS+ vs IMP+, each containing threshold and laterality-specific maps.

Signatures were examined at the hemisphere (3 signatures; left, right, or bilateral), threshold (6 p-values; *≤* 0.05/.01/.005/.001/.0005/.0001), and group comparison levels (3 comparisons; CS- vs CS+, CS+ vs IMP+, and CS- vs IMP+) for a total of up to 54 possible cortical signatures, depending on what thresholds, hemispheres, and group comparisons produced significant results.

**1.3 Identifying the Ideal Cortical Signature**

To determine the cortical signature map that best differentiated groups, ROC analyses were conducted. The area under the curve (*AUC*) for each comparison were compared using the pROC package^3^ (v1.18.0) within the R environment^4^ (v4.2.2). *AUC*s track the sensitivity and specificity of a variable at every potential threshold across the range of supplied values and measure the area under the generated curve to quantify how well the measure differentiates groups. *AUC* values range from 0 to 1, with 0.5 indicating random group designation and 1 indicating perfect separation between groups. *AUC* values between 0.7 and 0.8 are considered acceptable, 0.8 to 0.9 as excellent, and 0.9 to 1 as outstanding.^5^ ROC analyses were conducted for each cortical signature, evaluating their ability to differentiate CS- from CS+, CS+ from IMP+, and CS- from IMP+ groups, even if the cortical signature was derived from comparisons of a different pair of groups. Additionally, Cohen's *d* effect sizes were calculated to compare group differences identified by the ROC analyses. Significance for the effect sizes was determined using independent sample *t*-tests. To account for multiple comparisons, a false discovery rate (FDR) correction was applied across all cortical maps within each group difference comparison. Separate corrections were performed for the left hemisphere, right hemisphere, and the weighted combination of both hemispheres for CS- vs CS+, CS+ vs IMP+, and CS- vs IMP+ comparisons.

The *AUC* and *t*-tests were also repeated using subcortical brain volumes generated by FreeSurfer. To account for multiple comparisons, an FDR correction was also applied. The subcortical volumes were corrected for intracranial volume (ICV) using previously described methods.^6^ Similar to the cortical signatures analyses, 20% of the CS- sample was withheld to ensure parity in the subcortical volume analyses. The best AUCs for each comparison in subcortical and cortical regions were compared using pROC’s^3^ roc.test function.

The overall spatial correlation between DSAD and ADAD were tested in a spin test with 1000 permutations.^7^

**2 Results**

**2.1 Identifying the Ideal Cortical Signature for Each Group Comparison**

**2.1.1 Generating Cortical Signatures**

Regions that were significant after vertex and cluster correction for each group comparison were considered as potential cortical signatures, with separate signatures for each hemisphere (and their weighted average), vertex threshold, and group comparison. Significantly thinner cortices were observed within posterior brain regions (parietal, temporal, and lateral occipital lobes) in IMP+ compared to CS- (Supplemental Figure 1), but only in the right hemisphere at the most restrictive vertex threshold. No significant differences in cortical thickness were observed between CS- and CS+ at any threshold, following cluster correction. Significant differences were observed when comparing CS+ and IMP+, largely overlapped with the regions seen for the CS- and IMP+ comparison, though none survived thresholds below p ≤ 0.005. As vertex-wise thresholds became more stringent, the significant clusters were narrowed to the left medial and right lateral parietal lobes. These group, threshold, and cluster maps form a total of 25 cortical signatures (6 right, 5 left, and 5 bilateral for CS- vs IMP+, none for CS- vs CS+, and 3 right, 3 left, and 3 bilateral for CS+ vs Imp+) that were tested for their ability to differentiate groups. Clusters displaying differences in cortical thickness are depicted in Supplemental Figure 1.2.

**2.1.2 Selecting the Ideal Cortical Signature(s)**

The ideal cortical signature for each group comparison was identified based on AUC. Cortical signature AUCs for CS- vs IMP+ varied from poor (<0.7) to excellent (0.8-0.9) in differentiating CS- from CS+ (peak AUC = 0.83) and CS+ from IMP+ (peak AUC = 0.805). Excellent differentiation was achieved for CS- and IMP+ (peak AUC = 0.960). Figure 2B displays the ROC curves for each comparison for the regions that best differentiated groups. The ROC curves show the relatively diffuse (p ≤ 0.005) right hemisphere cortical signature differentiated CS- from CS+ (solid green line) better than CS+ from IMP+ (dashed orange line) while the reverse was seen for the more focal (p ≤ 0.0005) bilateral cortical signature. CS- from IMP+ (dotted purple line) had better AUCs than other comparisons.

Amyloid positivity (CS- vs CS+) and overall AD pathology (CS- vs IMP+) was best differentiated with relatively diffuse changes within the right fusiform and medial and lateral parietal regions (red clusters in the right hemisphere in Figure 1A, p ≤0.005, generated from the vertexwise comparison of CS- and IMP+). Relatively focal clusters in the left precuneus and right superior/inferior parietal best differentiated cognitive impairment (CS+ from IMP+; bilateral green clusters at p ≤0.0005 in Figure 1B, also generated from the vertexwise comparison of CS- and IMP+). While the bilateral map for the CS+ vs IMP+ comparison at p < .01, generated from the vertexwise comparison of CS+ to IMP+, had a higher AUC (.863) than the cortical signature identified for the comparison between CS+ and IMP+, this value was obtained from analyses that were biased due to the inclusion of the same subjects in the creation and testing of the cortical signatures. When tested with the same exclusion criteria applied to the other set of analyses, the AUC was not significantly higher (p = .843).

**2.2 Spatial Correlation Analyses**

**2.2.1 Down Syndrome Group Comparisons**

When comparing the effect size maps for CS- to CS+ and CS+ to IMP+, we observed that they were not correlated with each other (*Rho*s of 0.02 and 0.12, respectively). This finding was surprising since both effect maps were correlated with the effect size map for CS- vs IMP+ (average *Rho*s of 0.625 for CS- vs CS+ and 0.70 for CS+ vs IMP+).

**2.2.2 Down Syndrome and ADAD Comparisons**

The spatial correlation analyses revealed a high degree of similarity between the right and left hemispheres for DSAD and ADAD (*Rho* 0.548 vs *Rho* of 0.4). The overall spatial correlation between DSAD and ADAD (*Rho* 0.429) was significant (*p* < 0.001) after accounting for spatial autocorrelation.^7^ However, the pattern of change was more similar across the two hemispheres in ADAD (*Rho* 0.412) compared to DSAD (*Rho* 0.229) due to greater laterality for affecting the right hemisphere in DSAD.

Supplemental References

1. Su Y, D’Angelo GM, Vlassenko AG, et al. Quantitative analysis of PiB-PET with FreeSurfer ROIs. *PLoS One*. 2013;8(11):e73377. doi:10.1371/journal.pone.0073377

2. Su Y, Flores S, Hornbeck RC, et al. Utilizing the Centiloid scale in cross-sectional and longitudinal PiB PET studies. *Neuroimage Clin*. 2018;19:406-416. doi:10.1016/j.nicl.2018.04.022

3. Robin X, Turck N, Hainard A, et al. pROC: Display and Analyze ROC Curves. Published online November 1, 2023. Accessed June 5, 2024. https://cran.r-project.org/web/packages/pROC/index.html

4. R Core Team. R: A Language and Environment for Statistical Computing. Published online 2024. https://www.R-project.org/

5. Mandrekar JN. Receiver operating characteristic curve in diagnostic test assessment. *J Thorac Oncol*. 2010;5(9):1315-1316. doi:10.1097/JTO.0b013e3181ec173d

6. Buckner RL, Head D, Parker J, et al. A unified approach for morphometric and functional data analysis in young, old, and demented adults using automated atlas-based head size normalization: reliability and validation against manual measurement of total intracranial volume. *Neuroimage*. 2004;23(2):724-738. doi:10.1016/j.neuroimage.2004.06.018

7. Alexander-Bloch AF, Shou H, Liu S, et al. On testing for spatial correspondence between maps of human brain structure and function. *NeuroImage*. 2018;178:540-551. doi:10.1016/j.neuroimage.2018.05.07
